# Prevalence of hypertension and correlation with mental health in women with burning mouth syndrome: A case-control study

**DOI:** 10.3389/fcvm.2022.969148

**Published:** 2023-01-20

**Authors:** Federica Canfora, Elena Calabria, Giuseppe Pecoraro, Stefania Leuci, Noemi Coppola, Cristina Mazzaccara, Francesca Spirito, Massimo Aria, Luca D'Aniello, Michele Davide Mignogna, Daniela Adamo

**Affiliations:** ^1^Department of Neuroscience, Reproductive Sciences and Dentistry, University of Naples Federico II, Naples, Italy; ^2^Department of Molecular Medicine and Medical Biotechnology, University of Naples Federico II, Naples, Italy; ^3^CEINGE Advanced Biotechnologies, Naples, Italy; ^4^Department of Clinical and Experimental Medicine, University of Foggia, Foggia, Italy; ^5^Department of Economics and Statistics, University of Naples Federico II, Naples, Italy; ^6^Department of Social Sciences, University of Naples Federico II, Naples, Italy

**Keywords:** burning mouth syndrome (BMS), hypertension, women, chronic orofacial pain, cardiovascular risk factor

## Abstract

**Background:**

The relationship between hypertension (HTN) and chronic pain is still a matter of debate, and its prevalence in patients with burning mouth syndrome (BMS) has never been evaluated. This study aimed to assess the prevalence of HTN in women with BMS and to evaluate its relationship with potential predictors such as risk factors for cardiovascular diseases, pain, and mental health status analyzing differences with healthy women.

**Methods:**

In total, 250 women with BMS (WBMS) were prospectively recruited and compared with an equal number of healthy women (HW) matched for age. Education, body mass index, smoke and alcohol consumption, intensity and quality of pain, and psychological profile were further investigated to identify the potential predictors of HTN. Specifically, pain assessment [the Numeric Rating Scale (NRS) and Short-Form McGill Pain Questionnaire (SF-MPQ)] and psychological assessment [Hamilton Rating Scale for Depression and Anxiety (HAM-D and HAM-A), Pittsburgh Sleep Quality Index (PSQI), and Epworth Sleepiness Scale (ESS)] was carried out for the participants.

**Results:**

HTN was found in 128 (51.2%) WBMS and 76 (30.4%) HW (*p* < 0.001^**^). The scores of the NRS, SF-MPQ, HAM-D, HAM-A, and PSQI were statistically significantly higher in the WBMS than in the HW (*p* < 0.001^**^). A strongly linear correlation between HTN and employment status, systemic diseases, and education level (*p* < 0.001^**^) was found in WBMS, while a strong correlation between HTN and employment status, hypercholesterolemia, systemic diseases, and drug consumption was found in HW (*p* < 0.001^**^). No statistically significant correlation was found between HTN and pain, anxiety, depression, and sleep disturbances.

**Conclusion:**

These results suggest that WBMS showed a higher prevalence of HTN compared with controls. Unemployed WBMS with lower education and other systemic comorbidities are at an increased risk of developing HTN. HTN is associated with alteration in the vascular structure and function of the brain, and these processes accelerate brain aging, which contributes to a reduction in intracortical connectivity, thus affecting the modulatory system of control of pain in patients with BMS, independently of their mental health assessment. Predictors that may underlie this association remain unclear, taking into account the differences found in HW, and should be further elucidated.

## Introduction

Hypertension (HTN) is the leading cause of cardiovascular disease, and it is responsible for 8.5 million premature deaths from stroke, ischemic heart disease, and kidney disease worldwide (1–3). In addition, HTN is an evidence-based risk factor for brain aging and dementia (4, 5).

The number of people affected by HTN aged 30–79 years has doubled from 1990 to 2021, reaching 626 and 652 millions of women and men, respectively, in the world (6). Gender disparity in the HTN epidemiology reveals differences in age stratification; indeed, the prevalence of HTN in patients aged between 18 and 29 years is 3% in women vs. 8.5% in men, while in patients aged between 30 and 44 years, it is 7.3% in women vs. 15.8% in men (7). In contrast, this prevalence increases strongly after menopause, and it is more common in women than in men in the elderly population above the age of 75 years, reaching 78% (8).

The mechanisms in which sex interacts with vascular aging and subsequently with an increase in blood pressure are complex, including a multitude of hormonal, chromosomal, or even psychosocial factors (9). Indeed, sex steroids and the receptors through which they act are emerging as important mediators in the promotion and maintenance of sexual divergence in blood pressure regulation across the lifespan (10).

Obesity, dyslipidemia, impaired fasting glucose, and chronic pain are the most frequent comorbidity associated with HTN (11–14).

Burning mouth syndrome (BMS) is a chronic orofacial pain disorder with a strong female predilection; it is characterized by a generalized or localized intraoral burning or dysesthetic sensation or pain of the oral mucosa without any evidence of any specific mucosal lesions and/or laboratory findings (15). The overall prevalence of BMS was 1.73% in the general population and 7.72% in the clinical settings of dental practice with an average of 4%, reaching a prevalence of 18% in postmenopausal women (16). Several studies reported a consistent gender difference associated with BMS (16). Nasri-Heir et al. (17) reported the highest prevalence in women of middle age (>50 years) with a female/male ratio of 7:1, whereas in the recent meta-analysis by Wu et al. (16), the female/male ratio reported was 3:1. Possible factors behind these gender differences could include genetic factors affecting pain vulnerability as well as hormonal and psychosocial factors (18).

The pathophysiology of BMS includes central nervous system dysfunctions, which increase the central pain sensitization processes and reduce the functioning of descending pain inhibitory mechanisms (17). The higher pain sensitivity in women is probably due to biological sex differences in the ascending and descending modulation pathways and also for psychological phenomena that predominantly affect women (19).

While functional interactions between the pain inhibitory mechanism and the cardiovascular system exist (20), blood pressure is consistently and inversely associated with pain perception in chronic pain-free subjects. Indeed, elevated blood pressure may determine the attenuation of acute pain sensitivity (blood pressure-related hypoalgesia), and presumably, a similar phenomenon should be attended also in chronic pain status (20). However, recent studies found that, in patients with chronic pain, the relationship between blood pressure and pain sensitivity is completely reversed, and consequently, higher blood pressure has been associated with increased or higher sensitivity in the perception of chronic pain intensity (14, 20, 21).

Based on the above studies (14, 20, 21) and taking into account the positive relationship between elevated blood pressure and impaired pain perception, we assumed a possible association between HTN and a condition of chronic orofacial pain such as BMS. In addition, several studies underline that changes in hormonal profile and psychological factors during menopause could have a role in the development of both conditions in women (9, 22–24).

No published studies have examined the prevalence of HTN in the BMS population, specifically in women who are the most frequently affected population.

Therefore, this study aimed to investigate the prevalence of HTN in a wide sample of women with BMS (WBMS) compared with a control group of healthy women (HW) matched for age and to identify the potential predictors of HTN in WBMS and HW, analyzing the differences between the two groups and taking in account sociodemographic profile (age, employment, marital status), body mass index (BMI), risk factors (smoking and alcohol use), other systemic comorbidities and drug consumption, pain evaluation, and psychological factors.

## Materials and methods

### Study design and participants

This was an observational case-control study that was conducted between April 2020 and January 2022 at the Oral Medicine Department of the University of Naples “Federico II” in accordance with the ethical principles of the World Medical Association Declaration of Helsinki. It was approved by the Ethical Committee of the University (Approval Number: 251/19—the date of approval was February 20, 2019). The adopted methods conformed with the Strengthening of the Reporting of Observational Studies in Epidemiology (STROBE) guidelines for observational studies (25).

**Figure 1:**
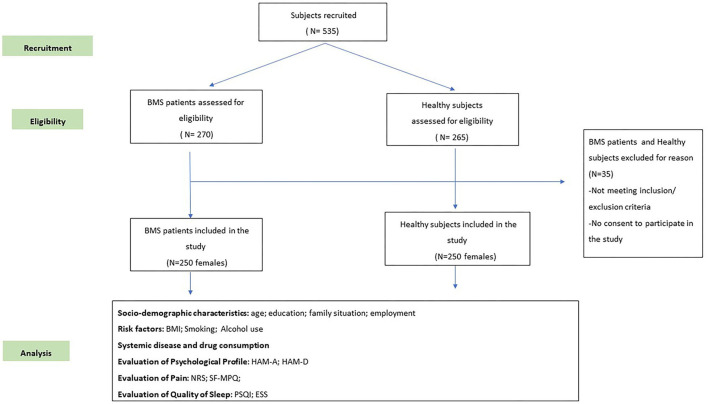
A flow chart of the study. BMS, burning mouth syndrome; BMI, Body Mass Index; ESS, Epworth Sleepiness Scale; HAM-A, Hamilton Rating Scale for Anxiety; HAM-D, Hamilton Rating Scale for Depression; NRS, Numeric Rating Scale; PSQI, Pittsburgh Sleep Quality Index; SF-MPQ, Short-Form McGill Pain Questionnaire.

At the baseline appointment (time 0), 270 patients in the study group and 265 individuals in the control group were considered eligible for this study. However, only 250 individuals in each group met the inclusion and exclusion criteria (Figure 1). All the participants prospectively recruited were women aged at least 18 years. The case group included patients suffering from BMS at the first consultation, which referred to the BMS symptom onset antecedent to any new drugs introduced in their treatment to exclude any causative effect, as described in previous studies (26–28). The control group included healthy subjects presenting at the hospital during the study period for dental treatments. Every subject considered eligible has been included in this study after having provided written informed consent. No payment was provided for participation. The patients and controls were matched by age. First, we recruited the patients and calculated their average age; then, we recruited the controls to obtain a matched sample.

In accordance with the International Classification of Orofacial Pain (ICOP 2020) 1st edition (15), the inclusion criteria of the WBMS group were as follows:

- Female patients aged at least 18 years;
- Patients experiencing an intraoral burning or dysesthetic sensation, recurring daily for more than 2 h per day for more than 3 months, without evident causative lesions on clinical examination and investigation; the pain has the characteristics of burning quality and is experienced superficially in the oral mucosa;
- Patients with normal blood test findings (including blood count, blood glucose levels, glycated hemoglobin, serum iron, ferritin, and transferrin); and
- Patients who are not currently in treatment with psychotropic drugs.

The WBMS group exclusion criteria were as follows:

- Patients suffering from diseases that could be recognized as a causative factor of BMS,
- Patients unable to understand or complete the questionnaires,
- Patients having a history of a psychiatric disorder or a neurological or organic brain disorder,
- Patients undergoing treatment with psychotropic drugs or systemic drugs possibly associated with oral symptoms,
- Patients having a history of alcohol or substance abuse, and
- Patients suffering from obstructive sleep apnoea syndrome (OSAS).

The inclusion criteria of the HW were as follows:

- Female subjects aged at least 18 years,
- Subjects without any lesion of the oral mucosa,
- Subjects without psychiatric disorder or a neurological or organic brain disorder,
- Subjects without a history of BMS,
- Subjects with normal blood test findings (including blood count, blood glucose levels, glycated hemoglobin, serum iron, ferritin, and transferrin), and
- Subjects who had not undergone treatment with psychotropic drugs.

The exclusion criteria of the HW were as follows:

- Subjects unable to understand or complete the questionnaires,
- Subjects having a history of alcohol or substance abuse,
- Subjects suffering from OSAS.

### Procedure

In the course of routine initial clinical evaluation, all the patients underwent a careful medical analysis, specifically an intra- and extra-oral examination by a board-certified expert clinician in oral medicine (DA). The patients had subsequently been assessed with regard to oral symptoms and the sites involved, age, years of education, family situation, job status, risk factors (current smoking status and alcohol consumption), medical comorbidities, and systemic drugs taken. The blood pressure (BP) has been recorded after the patient was seated for a minimum of 5 min in a standardized fashion during each examination cycle (29). The BP was calculated as the mean of two measurements recorded by a physician. We defined hypertension as having systolic blood pressure of 140 mmHg or greater, diastolic blood pressure of 90 mmHg or greater, or taking medication for hypertension (30). Moreover, we used measured weight and height to calculate the body mass index (BMI) as weight (kilograms) divided by the square of height (meters) (31). According to the WHO classification, the cutoff values considered were 18.5–24.9 kg/m^2^ for normal, 25.0–29.9 kg/m^2^ for overweight, and > 30 kg/m^2^ for obesity. In particular, obesity class I: BMI of 30–34.9 kg/m^2^, obesity class II: BMI of 35–39.9 kg/m^2^, obesity class III: BMI of ≥40 kg/m^2^ (also referred to as severe, extreme, or massive obesity) (32, 33).

### Pain and psychological profile assessment

A set of predefined questionnaires were administered to patients and controls to comprehensively analyze the intensity and quality of pain experienced, psychological profile, and sleep quality.

The Numerical Rating Scale (NRS) (34) and the Short Form of the McGill Pain Questionnaire (SF-MPQ) (35) were administered to evaluate the intensity and quality of pain of the sample group.

The NRS is an 11-point scale where the two endpoints are, respectively, the extremes of no pain and worst pain (34). This could be graphically administered, through a linear 11-box scale, or verbally administered. The SF-MPQ (35) is a multidimensional pain questionnaire that measures the quality of pain. This scale has 15 items considering the sensory, affective, and evaluative aspects of the perceived pain (34). Each item scored from 0 (none) to 3 (severe). There are no established critical cutoff points for the interpretation of the scores, and a higher score indicates worse pain.

The Hamilton Depression Rating Scale (HAM-A) (36) and the Hamilton Rating Scale for Anxiety (HAM-D) (37) were administered to evaluate anxiety and depression symptoms, respectively.

The HAM-A is a clinician-administered anxiety assessment scale containing 14 items, scored from 0 to 4, which evaluate both somatic anxiety and psychic anxiety. A total score of <17 indicates mild severity, a score between 18 and 24 indicates mild to moderate, and a score between 25 and 30 indicates moderate to severe (37).

The HAM-D is a hetero-administered scale containing 21 items that explore the affective field scoring from 0 to 54. The cutoff score considered are as follows: a score between 7 and 17 indicates mild depression, a score between 18 and 24 indicates moderate depression, and a score of >24 indicate severe depression. The HAM-A is a clinician-administered anxiety assessment scale containing 14 items, scored from 0 to 4, which evaluates both somatic anxiety and psychic anxiety. A total score of <17 indicates mild severity, a score between 18 and 24 indicates mild to moderate severity, and a score of 25–30 indicates moderate to severe severity (38).

The daytime sleepiness and the subjective sleep quality were evaluated using the Epworth Sleepiness Scale (ESS) (39) and the pittsburgh sleep quality index (PSQI) (40), respectively. First, the ESS evaluates the sleep propensity in daily life through 8 items, each scored from 0 to 3. On this scale, a higher score corresponds to higher daytime sleepiness (41, 42). Second, the PSQI considers a period of 1 month to evaluate sleep quality, evaluating seven components each scored from 0 to 3: subjective sleep quality, sleep latency, sleep duration, habitual sleep efficiency, sleep disturbances, use of sleeping medication, and daytime dysfunction (43). PSQI total score ranges between 0 and 21, and a higher score corresponds to the worst sleep quality (44).

### Statistical analysis

The statistical analysis was performed using the R software (v. 4.2.0 – R Core Team, 2016) (45). Descriptive statistics, including means, standard deviations (SDs), medians, and interquartile ranges (IQRs), were measured to summarize the sociodemographic and clinical characteristics of the WBMS and HW.

Fisher's exact test was used to assess the significant differences between frequencies for systematic diseases, drug consumption, antihypertensive drugs, oral symptoms, sites involved, and clinical parameters (psychological profile, and sleep and pain assessment) between WBMS and HW and between WBMS with and without hypertension, while the Mann–Whitney U test was computed for comparing median values.

Dependence analysis among WBMS and HW with HTN and qualitative predictors was performed. A significant difference between frequencies was measured by Fisher's exact test.

Dependence analysis among WBMS and HW with HTN and quantitative predictors was performed. Differences between groups were tested with the Mann–Whitney U test comparing median values. In all analyses, the Bonferroni correction was used to counteract the multiple comparisons problem.

## Results

The demographic variables and the risk factors are shown in Table 1. WBMS reported a statistically significantly lower education level (in years) and a higher level of unemployment compared with the HW (*p* < 0.001^**^). With respect to the family and marital status, a statistically significantly higher proportion of HW was divorced (*p* = 0.013^*^) in comparison to the WBMS. Additionally, WBMS presented a statistically significant higher percentage of heavy smokers (>15 cigarettes, 25 subjects-10%; *p* = 0.003^**^), while, overall, WBMS consumed less alcohol as there were statistically significantly more non-habitual alcohol users (*p* = 0.016^*^) compared to the control group. The frequency distributions of participants depending on the BMI categories revealed that overall, WBMS showed a considerably higher BMI than HW (*p* = 0.001^**^), especially with regard to the overweight and class I obesity categories.

**Table 1:** Socio-demographic profile and risk factors of 250 WBMS patients and 250 HW.

**Demographic variables**	**WBMS**	**HW**	***P*-value**
**Age** (in years)	**Mean** **±SD** 62.3 ± 11.4	**Mean** **±SD** 60.8 ± 11.7	0.146
**Education** (in years)	**Mean** **±SD** 9.2 ± 4.55	**Mean** **±SD** 11.4 ± 4.81	< 0.001^**^
**Family situation** - Single - Married - Divorced - Widowed	**Frequency (%)** 23 (9.2) 195 (78) 8 (3.2) 24 (9.6)	**Frequency (%)** 29 (11.6) 182 (72.8) 22 (8.8) 17 (6.8)	0.367 0.255 0.013^*^ 0.317
**Employment** - Employed - Unemployed	**Frequency (%)** 68 (27.2) 182 (72.8)	**Frequency (%)** 106 (42.4) 144 (57.6)	< 0.001^**^ < 0.001^**^
**Risk factors**	**Frequency (%)**	**Frequency (%)**	***P*** **-value**
**Smoking**			
- Never - < 5 cigarettes - 5–10 cigarettes - 10–15 cigarettes - >15 cigarettes	186 (74.4) 10 (4) 11 (4.4) 18 (7.2) 25 (10)	198 (79.2) 18 (7.2) 13 (5.2) 13 (5.2) 8 (3.2)	0.244 0.172 0.835 0.459 0.003^**^
**Alcohol use**			
- Never - Yes (1 unit) - Yes (2 units) - Yes (>2)	223 (89.2) 22 (8.8) 5 (2) 0 (0)	203 (81.2) 40 (16) 6 (2.4) 1 (0.4)	0.016^*^ 0.020^*^ 1.000 1.000
**Body Mass Index (kg/m2)**			
- BMI < 18.5 - BMI: 18.5-24.9 *normal* - BMI: 25.0-29.9 *overweight* - BMI: 30-34 *class I obesity* - BMI: 35-39.99 *class II obesity* - BMI>40 *class III obesity*	1 (0.4) 69 (27.6) 147 (58.8) 28 (11.2) 2 (0.8) 3 (1.2)	7 (2.8) 133 (53.2) 90 (36) 18 (7.2) 2 (0.8) 0 (0)	< 0.001^**^
**BMI**	**Mean** **±SD** 26.8 ± 3.70	**Mean** **±SD** 24.5 ± 3.58	

The significance difference between means was measured by the student t-test.

* Significant 0.01 < p ≤ 0.05,

** Significant p ≤ 0.01.

The significance difference among the percentages was measured by the Fisher's exact test. BMI, Body Mass Index; WBMS, women with burning mouth syndrome; HW, healthy women.

The prevalence of systemic disease and drug consumption are summarized in Table 2. A statistically significantly higher proportion of WBMS presented HTN and hypercholesterolemia compared to HW (*p* < 0.001^*^), while no significant differences were found with respect to all the other comorbidities. In detail, 128 (51.2%) WBMS and 76 (30.4%) HW showed HTN (*p* < 0.001^**^); similar results were present for hypercholesterolemia affecting 90 (36%) WBMS and 53 HW (21.2%; *p* < 0.001^**^).

**Table 2:** Prevalence of systemic diseases, drug consumption and antihypertensive drugs evaluation in 250 WBMS patients and 250 HW.

**Systemic diseases**	**WBMS** ** Frequency (%)**	**HW** ** Frequency (%)**	***P*-value**
Hypertension	128 (51.2)	76 (30.4)	< 0.001^**^
Hypercholesterolemia	90 (36)	53 (21.2)	< 0.001^**^
Hypothyroidism	46 (18.4)	34 (13.6)	0.179
Gastroesophageal reflux disease	36 (14.4)	28 (11.2)	0.349
Other cardiovascular disease	15 (6)	14 (5.6)	0.851
Neoplastic diseases	11 (4.4)	19 (7.6)	0.187
Asthma	10 (4)	8 (3.2)	0.811
HCV infection	6 (2.4)	4 (1.6)	0.751
Hyperthyroidism	5 (2)	4 (1.6)	1.000
Neurological disorders	5 (2)	3 (1.2)	0.724
Myocardial Infarction	4 (1.6)	6 (2.4)	0.751
Endocrine Disease	3 (1.2)	5 (2)	0.724
HBV infection	1 (0.4)	2 (0.8)	1.000
Others	47 (18.8)	45 (18)	0.908
**Drug consumption**	**WBMS** **Frequency (%)**	**HW** **Frequency (%)**	***P*** **-value**
Antiplatelets	62 (24.8)	22 (8.8)	< 0.001^**^
Proton pump inhibitors	50 (20)	29 (11.6)	0.014
Simvastatin	48 (19.2)	33 (13.2)	0.089
Beta blockers	42 (16.8)	38 (15.2)	0.715
ACE-inhibitors	41 (16.4)	21 (8.4)	0.009
Angiotensin II receptor antagonists (ARBs)	37 (14.8)	14 (5.6)	0.001^**^
Levothyroxine sodium	37 (14.8)	30 (12)	0.431
Thiazide Diuretics	28 (11.2)	20 (8)	0.229
Calcium Channel blockers	19 (7.6)	12 (4.8)	0.266
Blood thinner	6 (2.4)	9 (3.6)	0.602
Bisphosphonates	6 (2.4)	7 (2.8)	1.000
Steroids	4 (1.6)	2 (0.8)	0.686
**Antihypertensive drugs**	**WBMS** **Frequency (%)**	**HW** **Frequency (%)**	***P*** **-value**
Assumption- Yes	117 (46.8)	74 (29.6)	< 0.001^**^
1. Antihypertensive drug	75 (30)	48 (19.2)	0.007
2. Antihypertensive drugs	34 (13.6)	22 (8.8)	0.118
3. Antihypertensive drugs	8 (3.2)	3 (1.2)	0.221
4. Antihypertensive drugs	0 (0)	1 (0.4)	1.000

A significance difference between the percentages was measured by the Fisher's exact test.

^**^Significant with Bonferroni correction 0.002 for the systemic diseases.

^**^Significant with Bonferroni correction 0.003 for the drug consumption.

^**^Significant with Bonferroni correction 0.01 for antihypertensive drugs evaluation. HW, healthy women; WBMS, women with burning mouth syndrome.

**Figure 2:**
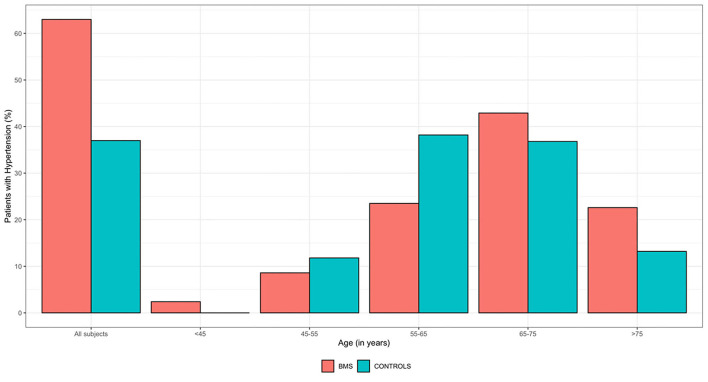
Frequency distribution of HTN by age ranges of 250 WBMS and 250 HW. HW, healthy women; WBMS, women with BMS.

As shown in Figure 2, the bar plot underlines the distribution of HTN considering the age stratification in which the highest percentage of WBMS with HTN (42.9%) is classified between the ages of 65 and 75 years, while for the HW, the highest percentage (38.2%) is between the ages of 55 and 65 years. Moreover, HTN was found in 29 WBMS (22.6%) and 10 HW (13.2%), aged >75 years.

**Figure 3:**
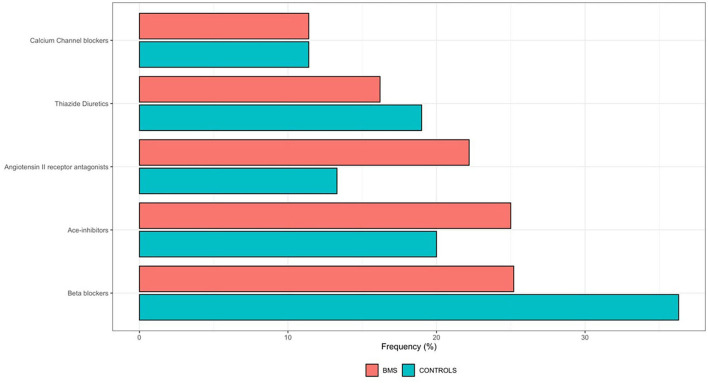
Frequency distribution of the antihypertensive drugs among 250 WBMS and 250 HW. HW, healthy women; WBMS, women with BMS.

Moreover, considering all the antihypertensive drugs, a statistically significantly higher proportion of WBMS (117; 46.8%) was on antihypertensive therapy compared to the HW (74; 29.6%) (*p* < 0.001^**^). The frequency distribution of the antihypertensive drugs among the WBMS is shown in Table 2 and Figure 3. In addition, the majority of WBMS (75; 30%) and HW (48; 19.2%) assumed only one antihypertensive drug, 35 WBMS (13.6%) assumed two antihypertensive drugs, 8 WBMS (3.2) assumed three antihypertensive drugs, and no WBMS assumed four antihypertensive drugs. On the contrary, only 11 out of 128 WBMS and 2 out of 76 HW were found to have HTN without assuming antihypertensive medications. A statistically significant difference was found in a higher percentage of WBMS (37; 14.8%) treated with the angiotensin II receptor antagonist (ARB) molecule compared to the HW (14; 5.6%) (*p* = 0.001^**^).

When comparing sociodemographic variables and risk factors between the subgroup of 128 WBMS with HTN and the subgroup of 122 WBMS without HTN, some differences were also detected (Supplementary Table 1). As expected, WBMS suffering from HTN were statistically older than those without HTN (67.2 ± 9.58 years vs. 51.1 ± 10.9 years) and had a lower education (8.03 ± 4.3 years vs. 10.4 ± 4.49 years) (*p* < 0.001^**^). In relation to the family status, there was a statistically higher prevalence of widowed (18; 14.1%) among the WBMS with HTN and a lower number of employed (18; 14.1%) (*p* < 0.001^**^). Differences were also detected with respect to the BMI as a statistically significantly higher proportion of WBMS with HTN (77; 602%) in comparison to the WBMS with no HTN (70; 57.4%) (*p* < 0.001). On the contrary, a higher percentage of WBMS without HTN showed a normal BMI (40; 32.8%) compared to the WBMS with HTN (29; 22.7%) (*p* < 0.001). In addition, as displayed in Supplementary Table 2, a comparison of the prevalence of systemic diseases and drug consumption between WBMS with and without HTN revealed no statistically significant difference, except from the antiplatelets, as a higher number of WBMS with HTN were under this medication (48; 37.5%) (*p* < 0.001^*^). Further information on the frequency distribution by age ranges of the total WBMS with and without HTN is displayed in Supplementary Figure 1 and Supplementary Table 3.

The type and location of the oral symptoms are shown in Table 3. Statistically significant differences were found between the cases and controls in relation to most of the symptoms. All the WBMS reported a burning sensation (250; 100%), which was the worst symptom reported, followed by xerostomia (151; 60.4%), dysgeusia (115; 46.2%), globus pharyngeus (103; 41.2%), intraoral foreign body sensation (60; 24%), sialorrhea (57; 22.8%), change in the tongue morphology (46; 18.4%), and itching (41; 16.4%). In contrast, halitophobia was the only symptom reported more in HW (19; 7.6%) than in WBMS (16; 6.4%) without any statistically significant difference. The most frequent sites involved were, in order, the tongue (225; 90%), followed by the anterior palate (163; 65.2%), the lips (162; 64.8%), the gums (154; 61.6%), and the cheeks (139; 55.6%).

**Table 3:** Prevalence of oral symptoms and sites involved in 250 WBMS patients and 250 HW.

**Oral symptoms**	**WBMS** **Frequency (%)**	**HW** >**Frequency (%)**	***P*-value**
Burning	250 (100)	14 (5.6)	< 0.001^**^
Xerostomia	151 (60.4)	34 (13.6)	< 0.001^**^
Dysgeusia	115 (46.2)	12 (4.8)	< 0.001^**^
Globus pharingeus	103 (41.2)	10 (4)	< 0.001^**^
Intraoral Foreign Body Sensation	60 (24)	14 (5.6)	< 0.001^**^
Sialorrhea	57 (22.8)	11 (4.4)	< 0.001^**^
Change in tongue morphology	46 (18.4)	1 (0.4)	< 0.001^**^
Itching	41 (16.4)	9 (3.6)	< 0.001^**^
Tingling sensation	32 (12.8)	6 (2.4)	< 0.001^**^
Occlusal Dysesthesia	22 (8.8)	5 (2)	0.001^**^
Diysosmia	17 (6.8)	3 (1.2)	0.002^**^
Halitophobia	16 (6.4)	19 (7.6)	0.726
Oral Dyskinesia	15 (6)	2 (0.8)	0.002^**^
**Sites involved**	**WBMS** **Frequency (%)**	**HW** **Frequency (%)**	***P*** **-value**
Tongue	225 (90.0)	33 (13.2)	< 0.001^**^
Anterior Palate	163 (65.2)	26 (10.4)	< 0.001^**^
Lips	162 (64.8)	31 (12.4)	< 0.001^**^
Gums	154 (61.6)	37 (14.8)	< 0.001^**^
Cheeks	139 (55.6)	32 (12.8)	< 0.001^**^
Floor of the mouth	123 (49.2)	27 (10.8)	< 0.001^**^
Soft Palate	116 (46.4)	25 (10)	< 0.001^**^

A significance difference between the percentages was measured by the Fisher's exact test.

^**^Bonferroni correction 0.004 for Oral Symptoms.

^**^Significant with Bonferroni correction 0.006 for Sites involved. HW, healthy women; WBMS, women with burning mouth syndrome.

Comparisons of the clinical parameters between the WBMS and HW are summarized in Table 4. A statistically significant difference was found in the NRS and SF-MPQ score between the two groups (*p* < 0.001^**^). The majority of WBMS (229; 91.6%) reported severe pain (NRS > 8) and the median and IQR of the SF-MPQ total score were 10 (7–12).

**Table 4:** Pain assessment, psychological profile and sleep in 250 WBMS patients and 250 HW.

**Clinical parameters**	**WBMS**	**HW**	***P*-value**
**NRS** - Mild pain 1–5 - Moderate pain 6–7 - Severe pain >8	**Frequency (%)** 6 (2.4) 15 (6) 229 (91.6)	**Frequency (%)** 238 (95.2) 7 (2.8) 5 (2)	< 0.001^**^
**SF-MPQ**	**Median; IQR** 10 [7–12]	**Median; IQR** 0 [0-0]	< 0.001^**^
**HAM-A** - Normal 0–7 - Mild severity 8–17 - Mild to moderate 18–25 - Moderate to severe 25–30	**Frequency (%)** 4 (1.6) 124 (49.6) 104 (41.6) 18 (7.2)	**Frequency (%)** 161 (64.4) 73 (29.2) 12 (4.8) 4 (1.6)	< 0.001^**^
**HAM-D** - Normal 0–7 - Mild depression 8–16 - Moderate depression 17–23 - Severe depression >24	**Frequency (%)** 3 (1.2) 116 (46.4) 102 (40.8) 29 (11.6)	**Frequency (%)** 169 (67.6) 60 (24) 16 (6.4) 5 (2)	< 0.001^**^
**PSQI** - PSQI total score < 5 - PSQI total score >5	**Frequency (%)** 24 (9.6) 226 (90.4)	**Frequency (%)** 117 (46.8) 133 (53.2)	< 0.001^**^
**ESS** - Normal range 0–10 - Mild sleepiness 11–14 - Moderate sleepiness 15–17 - Severe sleepiness >18	**Frequency (%)** 218 (87.2) 30 (12) 2 (0.8) 0 (0)	**Frequency (%)** 220 (88) 23 (9.2) 2 (0.8) 5 (2)	0.101

A significance difference between the percentages was measured by the Fisher's exact test. ^**^ Significant with Bonferroni correction 0.003. IQR is the interquartile range. The significance difference between medians was measured by the Mann–Whitney test. ^*^Significant 0.01 < p ≤ 0.05, ^**^Significant p ≤ 0.01. ESS, Epworth Sleepiness Scale; HAM-A, Hamilton rating scale for anxiety; HAM-D, Hamilton rating scale for depression; HW, healthy women; NRS, Numeric Rating Scale; PSQI, Pittsburgh Sleep Quality Index; SF-MPQ, Short-form McGill Pain Questionnaire; WBMS, women with burning mouth syndrome.

Statistically significant higher percentages of WBMS suffered from anxiety, depression, and sleep disturbances in comparison to the HW (*p* < 0.001^**^). Precisely, 246 WBMS (98.4%) and 89 HW (35.6%) showed anxiety (HAM-A>7), while 247 WBMS (98.8%) and 81 HW (32.4%) showed depression (HAM-D>7). In particular, the majority of WBMS (124; 49.6%) showed mild anxiety (HAM-A: 8–17), while 104 WBMS (41.6%) suffered from mild to moderate anxiety (HAM-A: 18–25). Regarding depression, 116 WBMS (46.4%) had mild depression (HAM-D: 8–18) and 102 WBMS (40.8%) had moderate depression (HAM-D: 17–23). Severe anxiety (HAM-A: 25–30) and severe depression (HAM-D>24) were found in 18 WBMS (7.2%) and in 29 WBMS (11.6%), respectively.

With respect to sleep evaluation, the WBMS showed a strongly statistically significant difference in the PSQI total score (*p* < 0.001^**^), as poor sleep (PSQI > 5) was found in 226 WBMS (90.4%) and in only 133 HW (53.2%), while no statistically significant difference was found in the ESS total score between the two groups (*p* = 0.101).

When comparing oral symptoms, the sites involved and the scores of pain (NRS, T-PRI), anxiety and depression (HAM-A, HAM-D), and sleep quality (PSQI, ESS), no differences were detected between WBMS with and without HTN (Supplementary Tables 4, 5).

A dependence analysis between HTN and qualitative and quantitative predictors was performed separately for WBMS and HW to analyze differences in the predictors of HTN between cases and controls. The results of the dependence analysis between HTN and qualitative and quantitative predictors in WBMS are summarized in Table 5. In detail, employment status was found to correlate with HTN (*p* < 0.001^**^); in particular, unemployed WBMS in this group were 110 (85.9%), while the employed WBMS suffering from HTN were only 18 (14.1%). Also, the systemic diseases were positively correlated with HTN (*p* < 0.001^**^), and specifically, WBMS suffering from diseases other than HTN were 124 (96.9%). Among the quantitative predictors, only education level, expressed in years, was found to correlate with HTN (*p* < 0.001^**^).

**Table 5:** Dependence analysis among 128 WBMS patients with HTN and qualitative and quantitative predictors.

**WBMS-qualitative predictors**	**HTN** **Frequency (%)**	***P*-value**
**Marital status**		
- Married - not married	97 (75.8) 31 (24.2)	0.545
**Employment**		
- Employed - Not employed	18 (14.1) 110 (85.9)	< 0.001^**^
**Smoking**		
- Smoker - No smoker	27 (21.1) 101 (78.9)	0.111
**Alcohol use**		
- Yes - No	12 (9.4) 116 (90.6)	0.542
**Hypercholesterolemia**		
- Yes - No	56 (43.8) 72 (56.2)	0.012
**Systemic diseases**		
- Yes - No	124 (96.9) 4 (3.1)	< 0.001^**^
**Drug consumptions**		
- Yes - No	96 (75) 32 (25)	0.074
**Quantitative Predictors**	**HTN** **Frequency(%)**	***P*** **-value**
NRS	10 [9.75–10]	0.360
SF-MPQ	10 [7.75–12]	0.339
HAM-A	18 [15–20.2]	0.245
HAM-D	18 [14–20]	0.502
PSQI	8 [8–9.25]	0.882
ESS	6.5 [5–9]	0.149
Education (in years)	8 [5–12.2]	< 0.001^**^
BMI (kg/m^2^)	26.9 [25.3–28.8]	0.073

A significance difference between the percentages was measured by the Fisher's exact test.

^**^Significant with Bonferroni correction 0.004 for qualitative predictors.

IQR is the interquartile range. The significance difference between medians was measured by the Mann–Whitney test.

^**^Significant with Bonferroni correction 0.006 for quantitative predictors.

BMI, Body Mass Index; ESS, Epworth Sleepiness Scale; HAM-A, Hamilton rating scale for anxiety; HAM-D, Hamilton rating scale for depression; NRS, Numeric Rating Scale; PSQI, Pittsburgh Sleep Quality Index; SF-MPQ, Short-form McGill Pain Questionnaire; WBMS, women with burning mouth syndrome.

The results of the dependence analysis between HTN and qualitative and quantitative predictors in HW are summarized in Table 6. The evaluation of qualitative predictors showed a correlation not only with employment status and systemic diseases (*p* < 0.001^**^) as in WBMS but also with hypercholesterolemia and drug consumption as in HW (*p* < 0.001^**^). No correlation was found between HTN and quantitative predictors in HW.

**Table 6:** Dependence analysis among 122 HW with HTN and qualitative and quantitative predictors.

**HWC- Qualitative Predictors**	**HTN** **Frequency (%)**	***P*-value**
**Marital status**		
- Married - Not married	57 (75) 19 (25)	0.887
**Employment**		
- Employed - Not employed	23 (30.3) 53 (69.7)	< 0.001^**^
**Smoking**		
- Smoker - No smoker	13 (17.1) 63 (82.9)	0.117
**Alcohol**		
- Yes - No	11 (14.5) 65 (85.5)	0.418
**Hypercholesterolemia**		
- Yes - No	21 (27.6) 55 (72.4)	< 0.001^**^
**Systemic diseases**		
- Yes - No	76 (100) (0)	< 0.001^**^
**Drug consumptions**		
- Yes - No	67 (88.2) 9 (11.8)	< 0.001^**^
**Quantitative predictors**	**HTN** **Frequency (%)**	***P*** **-value**
NRS	0 [0–0.25]	0.437
SF-MPQ	0 [0–1.25]	0.128
HAM-D	5 [2.75–9.25]	0.716
HAM-A	6 [3–11.2]	0.110
PSQI	6 (4–9)	0.010
ESS	6 (3–8)	0.149
Education (years)	10 [7.5–13]	0.011
BMI (kg/m^2^)	25.4 [22–26.9]	0.223

A significance difference between the percentages was measured by the Fisher's exact test.

^**^Significant with Bonferroni correction 0.003 for Qualitative Predictors.

IQR is the interquartile range. The significance difference between medians was measured by the Mann–Whitney test.

^**^Significant with Bonferroni correction 0.006 for quantitative predictors.

BMI, Body Mass Index; ESS, Epworth Sleepiness Scale; HAM-A, Hamilton rating scale for anxiety; HAM-D, Hamilton rating scale for depression; HW, healthy women; NRS, Numeric Rating Scale; PSQI, Pittsburgh Sleep Quality Index; SF-MPQ, Short-form McGill Pain Questionnaire.

## Discussion

Blood pressure and its regulatory systems have been proven to be deeply interconnected with pain modulation (20). For instance, both essential HTN and secondary HTN are effective in reducing acute pain perception throughout a process known as blood pressure-related hypoalgesia (46). On the contrary, in chronic pain sufferers, this mechanism seems to be under-regulated, and as a consequence, elevated blood pressure is associated with greater chronic pain intensity (14).

Nevertheless, the prevalence and role of HTN in chronic pain conditions are poorly understood, especially in women. The female's prevalence of HTN increases after menopause (>40 years), suggesting the pivotal role of sexual hormone imbalance in the pathophysiology of the disease (1, 10, 22). In detail, menopause promotes a derangement of the cardiocirculatory system with a marked decline in endothelium-dependent vasodilation and also in the emergence of other atherogenic factors (47, 48); in addition, the menopause transition broadly affects health and wellbeing in the midlife woman being associated with decreased physical activity and weight gain, impaired sleep, and negative mood (47, 49). All these aspects are also involved in the chronic pain experience, further causing an increase in blood pressure. Indeed, the fluctuations of estrogen hormones and, subsequently, their reduction influence the development and exacerbation of both HTN and pain (50, 51). This theory is supported by epidemiological studies in which the perimenopausal and postmenopausal women showed a higher prevalence of chronic pain and HTN compared to men (9, 52). Moreover, women showed lower pain threshold and tolerance, resulting in increased pain intensity (53). Additionally, it has been proven that women experiencing pain are less prone to use coping strategies, are predisposed to pain chronicization, and are more likely to seek the help of a pain specialist for treatment (50, 54).

BMS epidemiology highlights that it affects more female subjects, especially after menopause (16). Structural and functional alterations in the peripheral and central nervous system are considered in the etiopathogenesis of the disease, thus affecting the pain perception and predisposition for mood disorders, sleep disturbance, and cognitive impairment (17, 19, 55, 56), as suggested also in a recent study from Canfora et al. (57).

This is the first study that evaluated the prevalence of HTN in a wide sample of WBMS in comparison with an age-matched control group and explored the possible predictors of HTN in this disease. Finally, this study analyzed the potential role of HTN in the disease progression and the mutual interaction between HTN and pain, mood disorders, sleep, and other comorbidities.

The results of the study suggested a statistically significant difference in the prevalence of HTN in the sample with a higher prevalence of HTN in WBMS (51.2%) compared with HW (30.4%). Considering age stratification, the prevalence of HTN was higher in HW until 65 years but it increased in WBMS aged higher than 65 years. Precisely, the prevalence of HTN in HW with age < 65 years was in line with the epidemiological studies that investigate women's HTN in the general population (38.2%), and this percentage was found to be consistently lower in WBMS (23.5%).

Instead, this prevalence increased to 42.9% in WBMS aged between 65 and 75 years, resulting in a higher prevalence compared with the general prevalence of HTN in women and in patients suffering from chronic pain. Indeed, even if this prevalence is slightly higher compared with the study of Bruehl et al. (14) on 300 chronic pain patients (39%), these results support the possibility of the functional and overlapping links of the anti-nociceptive and the cardiovascular systems in which impairment in the mechanism of modulating both pain and blood pressure may increase HTN prevalence in WBMS (58–60).

Indeed, blood pressure is modulated by functional circuitry linking the hypothalamus, the nucleus tractus solitarius, the nucleus raphe magnus, and the rostral ventrolateral medulla in which the activity of central adrenergic fibers and alpha-2 receptors may prolong the activation of anti-nociceptive pathways in patients with BMS (61, 62).

Moreover, the bidirectional relationship between pain and blood pressure may involve the levels of cerebral catecholamine, as a result of changed catechol-O-methyltransferase (COMT)-dependent metabolism (63–65). Indeed, the gene which codes for this protein activity is highly polymorphic, and some variants contribute to a lower metabolism of norepinephrine to normetanephrine; the increase of these neurotransmitters not only contributes to higher blood pressure but also modifies pain response (66).

The impact of long-term elevated blood pressure on cerebral health involves structural pathological changes of the brain such as WMH, cortical thinning, enlarged Virchow-Robin spaces, and brain atrophy (67), suggesting that HTN can accelerate brain aging in the same areas involved in chronic pain. However, it is difficult to determine if these brain alterations are a direct consequence of HTN but it could explain the previous study results in which a high prevalence of WMH was found in patients with BMS (68, 69).

In this study, a statistically significant difference in years of education was found between WBMS and HW with lower educational attainment found in WBMS compared with HW, and it may be implicated in the impairment of blood pressure control and considered a predictor of HTN. These results are in line with previous studies' results, in which an increase in the year of education leads to an increase in the individual's knowledge and skills about the disease; on the contrary, unschooled patients were at greater risk of developing uncontrolled HTN (70, 71).

In addition, less educational attainment has a significant role also on the limited knowledge about healthcare and disease (72), such as BMS. For this reason, the patient's ability to manage both diseases is strictly dependent on their education (73).

Moreover, in this study, a higher prevalence of unemployment was found in WBMS (72.8%) compared with HW (57.6%), and this condition was a predictor of HTN as suggested from the results of the dependence analysis. This finding was in line with previous reports in which employment status was inversely associated with HTN in women (22, 74), maybe due to the employment's influence on a woman's socioeconomic status (75).

In line with previous studies, the coexistence of other systemic comorbidities represents a predictor of HTN in patients and in controls, and unfortunately, this is a non-modifiable risk factor of HTN (76, 77).

Therefore, unemployed WBMS with few years of education and other systemic comorbidities are at increased risk to develop HTN. Instead, the predictors of HTN are slightly different in HW group, showing that unemployment, systemic comorbidities, hypercholesterolemia, and drug consumption increase the risk of HTN.

In our sample, the common modifiable health risk behaviors of HTN such as tobacco use (78, 79) and alcohol consumption (80, 81) were not predictors of HTN in patients and controls, but probably these results have to be considered in light of the prevalence of non-smokers (74.4 and 79.9%, respectively) and no alcohol consumers (89.2 and 81.2%, respectively) among WBMS and HW.

In addition, in our sample, BMI was not a predictor of HTN but probably because the majority of WBMS and HW were not obese. In addition, disability related to diseases, such as BMS, makes patients more sedentary and prone to develop obesity. Therefore, body weight reduction in overweight women and the promotion of healthy lifestyle behaviors (50) represent an essential part of both HTN and BMS treatment.

From the analysis of the psychological profile, WBMS showed a higher prevalence of anxiety, depression, and sleep disturbance compared with HW, but no differences were found between WBMS with or without HTN.

Moreover, pain, anxiety depression, and sleep disturbances were not predictors of HTN both in WBMS and in HW. Therefore, the association between depression and anxiety and increased HTN risk remains inconsistent in this study in contrast with other studies that found depression associated with an increased (82, 83) or decreased (84) risk of HTN.

Patients receiving the HTN diagnosis could increase their psychological distress, which may further aggravate the adjunctive diagnosis of BMS (85, 86). Indeed, the coexistence of several medical comorbidities may have a labeling effect causing mental distress and a decrease in the quality of life, resulting in increased healthcare utilization (77).

Sleep disturbance (PSQI > 5) was found in 90.4% of WBMS, confirming the results of previous studies in which a high prevalence of poor sleep was found in patients with BMS (55, 87). Despite sleep disturbance not being considered a predictor of HTN in our sample, it is known that sleep disturbance is associated with an independent HTN risk (88, 89). Particularly, a 2016 American Heart Association (AHA) scientific statement concluded that there is strong epidemiological evidence that self-reported short sleep duration (<6 h) is a risk factor for HTN where women may be more prone to the effects of short sleep duration on HTN risk (89). This statement was confirmed by a review of 2012 in which higher HTN risk among short sleepers has been reported (90).

Short sleep may increase HTN risk through several physiological mechanisms, including disturbed autonomic balance, hormonal imbalances, inflammation and oxidative stress, greater predisposition to obesity, metabolic syndrome, and unhealthy lifestyle behaviors (88–90). Thus, when present simultaneously, BMS, HTN, mood disorder, and sleep disturbance represent a toxic combination that affects the quality of life of individuals, worsening the outcome of the disease (88, 91).

The results of this study highlight that BMS is a complex disease, with several intertwined comorbidities that may aggravate and prevent the healing of patients if a complete medical and psychological assessment and treatment of all conditions is not carried out. Therefore, despite dental professionals playing a central role in the diagnosis and the management of disease, it is crucial to improve the knowledge about BMS also among medical professionals and promote multidisciplinary collaboration to identify and treat the possible associated comorbidities and reduce the societal burden caused by BMS, further worsening the association with HTN and mood disorder.

## Conclusion

WBMS showed a higher prevalence of HTN compared with HW. Unemployed WBMS with lower education and other systemic comorbidities are at an increased risk to develop HTN. The mechanism whereby these phenomena are associated is not completely clear by the results of this study although it is reasonable to consider the interaction of the genetic, environmental, and biological factors that could contribute to both HTN and BMS development. Indeed, the effects of the cardiovascular sympathetic stimulation in response to the failure of pain-regulatory mechanisms may contribute to broadening pain perception and HTN. The association of BMS and HTN may, in turn, accelerate brain aging contributing to the occurrence of WMH, resulting in intracortical connectivity reduction, which further affects pain processing and produces a vicious circle.

Moreover, a higher prevalence of anxiety, depression, and sleep disturbance was found in WBMS compared with HW. Considering the deleterious effects of concomitant HTN and mood disorders, early recognition and proper treatment of both conditions in patients affected by BMS are important.

Healthy lifestyle behaviors, in addition to treatments, are essential in WBMS to promote psychological wellbeing, improve the quality of life, and prevent early brain aging. Further studies will be needed to confirm the association between HTN and BMS.

## Limitation

This study has some limitations. First, an important limitation of the study is related to the hypertension diagnosis because it was not possible to verify if hypertension preceded the chronic pain onset or, on the contrary, the chronic pain came first. This could be important in the etiopathogenesis interpretation. Second, the duration of antihypertensive drug assumption and eventual switching in the therapy could not be reported by the patients; as a consequence, we do not know if there were resistant hypertension among these patients; and consequently, although no correlation has been found with HTN and pain scores, it is not possible to address the question of whether controlling the high pressure may have a role in modulating pain perception.

## Data availability statement

The raw data supporting the conclusions of this article will be made available by the authors, without undue reservation.

## Ethics statement

The studies involving human participants were reviewed and approved by the Ethical Committee of the University of Naples Federico II (Approval Number: 251/19—the date of approval was February 20, 2019). The patients/participants provided their written informed consent to participate in this study.

## Author contributions

DA, FC, and MM: conceptualization. DA, FC, EC, GP, and MM: methodology. LD'A and MA: software and formal analysis. MM and DA: validation and supervision. FC, EC, SL, NC, CM, GP, FS, LD'A, MA, and MM: investigation. FC, EC, SL, NC, and MM: resources. FC, EC, CM, SL, NC, FS, LD'A, MA, and MM: data curation. FC, DA, and EC: writing—original draft preparation. FC, EC, LD'A, MA, GP, DA, and MM: writing—review and editing. DA, FC, EC, and MM: visualization. All authors have contributed to the work and are familiar with the primary data, each has read the final version of the manuscript, approved its content, and have agreed to have their name added to the paper.
