# Supplementary material for: Prevalence of hypertension and correlation with mental health in women with burning mouth syndrome: A case-control study
Source: Front Cardiovasc Med. 2023 Jan 20;9:969148. doi: 10.3389/fcvm.2022.969148 (PMC9894887; doi:10.3389/fcvm.2022.969148)
Supplement: Supplementary file 1 [file Data_Sheet_1.docx]

**Supplementary tables**

***Table 1S*. *Socio-demographic profile and risk factors of 128 WBMS patients with HTN and 122 WBMS patients without HTN***

| **Demographic variables** | **Yes HTN** | **No HTN** | **P-value** |
| --- | --- | --- | --- |
|  | **Frequency (%)** 128 (51.2) | **Frequency (%)**  122(48.8) |  |
| **Age** (in years) | **Mean ± SD**  67.2±9.58 | **Mean ± SD**  51.1±10.9 | **<0.001**** |
| **Education** (in years) | **Mean ± SD**  8.03±4.3 | **Mean ± SD**  10.4±4.49 | **<0.001**** |
| **Family situation**   - Single - Married - Divorced - Widowed | **Frequency (%)**  8 (6.2) 97 (75.8) 7 (5.5) 18 (14.1) | **Frequency (%)**  13 (10.7) 97 (79.5)  1 (0.8) 5 (4.1) | 0.257 0.545 0.067 **0.008**** |
| **Employment**   - Employed - Unemployed | **Frequency (%)**  18 (14.1)  110 (85.9) | **Frequency (%)**  50 (41) 72 (59) | **<0.001****  **0.001**** |
| **Risk factors** | **Frequency (%)** | **Frequency (%)** | **P-value** |
| **Smoking**   - Never - <5 cigarettes - 5-10 cigarettes - 10-15 cigarettes - >15 cigarettes | 101 (78.9) 5 (3.9)  2 (1.6) 8 (6.2) 12 (9.4) | 85 (69.7)  5 (4.1)  9 (7.4)  10 (8.2)  13 (10.7) | 0.111  1.000  **0.031***  0.629  0.834 |
| **Alcohol use**   - Never - Yes (1 unit) - Yes (2 units) - Yes (>2) | 116(90.6)  7 (5.5)  5 (3.9)  0 (0) | 107 (87.7)  15 (12.3) 0 (0)  0 (0) | 0.543  0.074  0.060  - |
| **Body Mass Index**   - BMI | **MEAN ± SD** 27.2±3.71 | **MEAN ± SD** 26.5±3.67 | 0.073 |

*The significance difference between means was measured by the t-student test.
* Significant 0.01 < p ≤ 0.05. ** Significant p ≤ 0.01.*

*The significance difference among the percentages was measured by the Fisher’s exact test.
* Significant 0.01 < p ≤ 0.05. ** Significant p ≤ 0.01.*

*Abbreviations: HTN: Hypertension; WBMS: women with burning mouth syndrome;*

**Table 2S. *Prevalence of systemic diseases, drug consumption and drugs consumption in 128 WBMS patients with hypertension and 122 WBMS patients without hypertension***

| **Systemic diseases** | **Yes HTN**  **Frequency (%)** | **No HTN**  **Frequency (%)** | **P-value** |
| --- | --- | --- | --- |
| Hypercholesterolemia | 56 (43.8) | 34 (27.9) | 0.012 |
| Hypothyroidism | 26 (20.3) | 20 (16.4) | 0.514 |
| Gastroesophageal reflux disease | 22 (17.2) | 14 (11.5) | 0.212 |
| Other cardiovascular disease | 9 (7) | 6 (5) | 0.598 |
| Asthma | 8 (6.2) | 2 (1.6) | 0.103 |
| Neoplastic diseases | 5 (3.9) | 6 (4.9) | 0.765 |
| Myocardial Infarction | 4 (3.1) | 0 (0) | 0.122 |
| HCV infection | 3 (2.3) | 3 (2.5) | 1.000 |
| Neurological disorders | 2 (1.6) | 3 (2.5) | 0.678 |
| Endocrine Disease | 1 (0.8) | 2 (1.6) | 0.615 |
| Hyperthyroidism | 1 (0.8) | 4 (3.3) | 0.204 |
| HBV infection | 0 (0) | 1 (0.8) | 0.488 |
| Others | 15 (11.7) | 32 (26.2) | 0.004 |
| **Drugs consumption** | **Yes HTN**  **Frequency (%)** | **No HTN**  **Frequency (%)** | **P-value** |
| Antiplatelets | 48 (37.5) | 14 (11.5) | **<0.001*** |
| Proton pump inhibitors | 31 (24.2) | 19 (15.6) | 0.113 |
| Simvastatin | 25 (19.5) | 23 (18.9) | 1.000 |
| Levothyroxine sodium | 20 (15.6) | 17 (13.9) | 0.725 |
| Blood thinner | 5 (3.9) | 1 (0.8) | 0.214 |
| Bifosfonati | 4 (3.1) | 2 (1.6) | 0.684 |
| Steroids | 1 (0.8) | 3 (2.5) | 0.360 |

*A significance difference between the percentages was measured by the Fisher’s exact test.*

*** Significant with Bonferroni correction 0.002 for the systemic diseases*

*** Significant with Bonferroni correction 0.003 for the drug consumption*

** Significant 0.01 < p ≤ 0.05. ** Significant p ≤ 0.01.*

*Abbreviation: HTN: Hypertension; WBMS: women with BMS*

**Figure 1S/Table 3S. *Frequency distribution by age-ranges of 128 WBMS with HTN and 122 WBMS without HTN.***

*
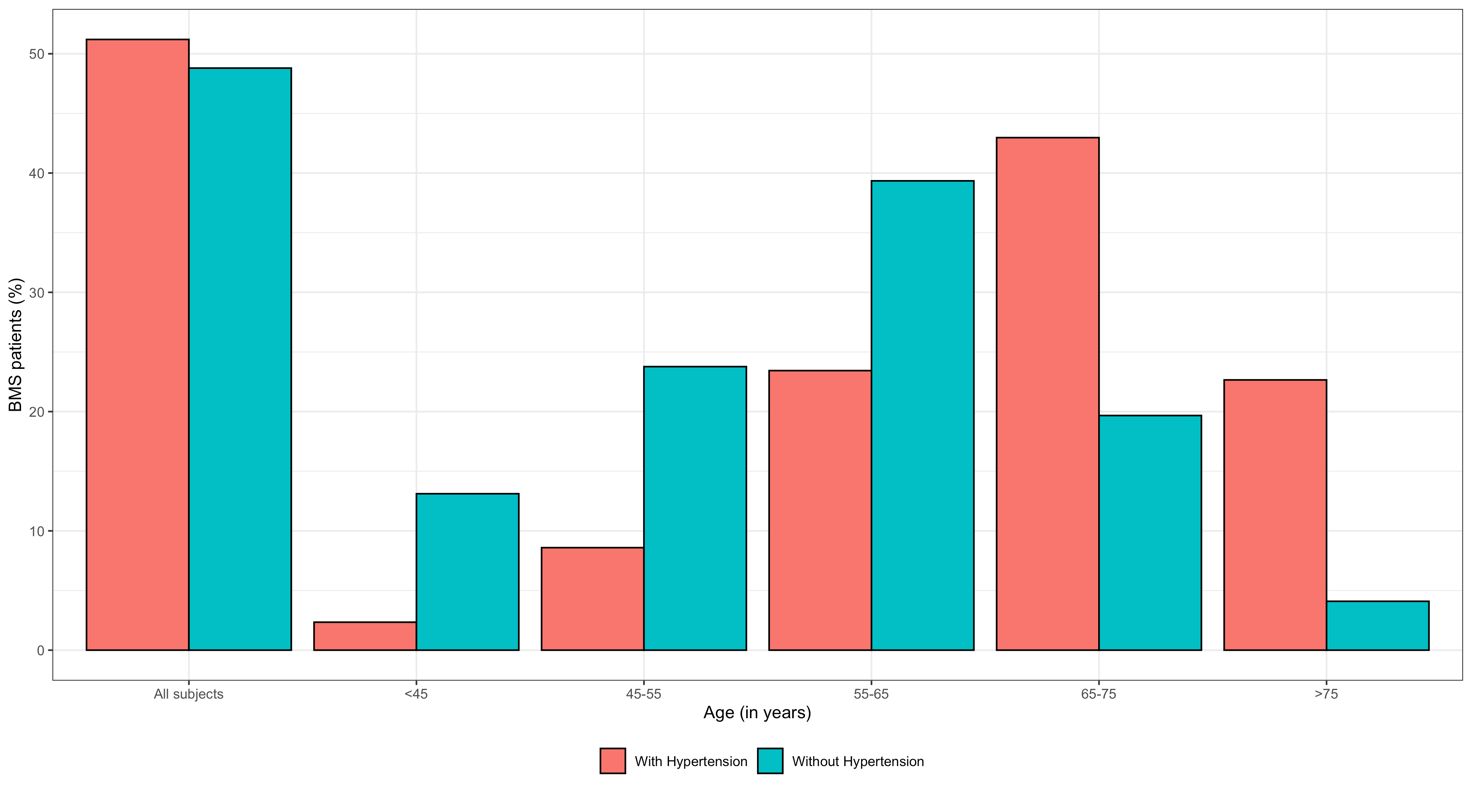
*

| **WBMS patients** | **Yes HTN** | **No HTN** |
| --- | --- | --- |
|  | **Frequency (%)** | **Frequency (%)** |
| **All subjects** | 128 (51.2) | 122 (48.8) |
| **Classification per Age** |  |  |
| <45 | 3 (2.3) | 16 (13.1) |
| 45-55 | 11 (8.6) | 29 (23.8) |
| 55-65 | 30 (23.4) | 48 (39.3) |
| 65-75 | 55 (43) | 24 (19.7) |
| >75 | 29 (22.7) | 5 (4.1) |

*Abbreviation: HTN: Hypertension; WBMS: women with BMS*

**Tab.4S *Prevalence of oral symptoms and sites involved in 128 WBMS patients with HTN and 122 WBMS patients without HTN***

| **Oral symptoms** | **Yes HTN**  **Frequency (%)** | **No HTN**  **Frequency (%)** | **P-value** |
| --- | --- | --- | --- |
| Burning | 117 (91.4) | 103 (84.4) | 0.119 |
| Xerostomia | 82 (64.1) | 69 (56.6) | 0.246 |
| Dysgeusia | 63 (49.6) | 52 (42.6) | 0.309 |
| Globus pharingeus | 56 (43.8) | 47 (38.5) | 0.442 |
| Intraoral Foreign Body Sensation | 30 (23.4) | 30 (24.6) | 0.883 |
| Sialorrhea | 27 (21.1) | 30 (24.6) | 0.548 |
| Itching | 22 (17.2) | 19 (15.6) | 0.737 |
| Change in tongue morphology | 22 (17.2) | 24 (19.7) | 0.628 |
| Tingling sensation | 17 (13.3) | 15 (12.3) | 0.852 |
| Occlusal Dysesthesia | 8 (6.2) | 14 (11.5) | 0.182 |
| Diysosmia | 8 (6.2) | 9 (7.4) | 0.804 |
| Oral Dyskinesia | 7 (5.5) | 8 (6.6) | 0.794 |
| Halitophobia | 6 (4.7) | 10 (8.3) | 0.306 |
| **Sites involved** | **Yes HTN**  **Frequency (%)** | **No HTN**  **Frequency (%)** | **P-value** |
| Tongue | 118 (92.2) | 107 (87.7) | 0.293 |
| Lips | 90 (70.3) | 72 (59) | 0.065 |
| Anterior Palate | 88 (68.8) | 75 (61.5) | 0.235 |
| Gums | 80 (62.5) | 74 (60.7) | 0.796 |
| Cheeks | 77 (60.2) | 62 (50.8) | 0.162 |
| Floor of the mouth | 68 (53.1) | 55 (45.1) | 0.209 |
| Soft Palate | 65 (50.8) | 51 (41.8) | 0.165 |

*The significance difference among the percentages was measured by the Fisher’s exact test.
* Significant 0.01 < p ≤ 0.05. ** Significant p ≤ 0.01.*

*Abbreviation: HTN: Hypertension; WBMS: women with BMS*

**Table 5S. *Pain assessment, psychological profile and sleep in 128 WBMS patients with HTN and 122 WBMS patients without HTN***

| **Clinical parameters** | **Yes HTN** | **No HTN** | **P-value** |
| --- | --- | --- | --- |
| **NRS**  - Mild pain 1-5  - Moderate pain 6-7  - Severe pain >8 | **Frequency (%)**  1 (0.8) 6 (4.7) 121 (94.5) | **Frequency (%)** 5 (4.1) 9 (7.4) 108 (88.5) | 0.165 |
| **SF-MPQ** | **Median; IQR** 10 [7.75-12] | **Median; IQR** 10 [7-12] | 0.339 |
| **HAM-A**  - Normal 0-7  - Mild severity 8-17  - Mild to moderate 18-25  - Moderate to severe 25-30 | **Frequency (%)**  3 (2.3) 56 (43.8) 60 (46.9) 9 (7) | **Frequency (%)**  1 (0.8) 68 (55.7) 44 (36.1) 9 (7.4) | 0.222 |
| **HAM-D**  - Normal 0-7  - Mild depression 8-16  - Moderate depression 17-23  - Severe depression >24 | 1 (0.8) 56 (43.8) 61 (47.7) 10 (7.8) | 2 (1.6) 60 (49.2) 41 (33.6) 19 (15.6) | 0.053 |
| **PSQI**  - <5  - poor sleep quality >5 | 13 (10.2) 115 (89.8) | 11 (9) 111 (91) | 0.831 |
| **ESS**  - Normal range 0-10  - Mild sleepiness 11-14  - Moderate sleepiness 15-17  - Severe sleepiness >18 | 112 (87.9) 15 (11.7) 1 (0.8) 0 (0) | 106 (86.9) 15 (12.3) 1 (0.8) 0 (0) | 1.000 |

*The significance difference among the percentages was measured by the Fisher’s exact test.
* Significant 0.01 < p ≤ 0.05. ** Significant p ≤ 0.01.*

*IQR is the interquartile range. The significance difference between medians was measured by the Mann–Whitney test.*

**Significant 0.01 < p ≤ 0.05, **Significant p ≤ 0.01.*

*Abbreviations*: ESS: Epworth Sleepiness Scale; HAM-A: Hamilton rating scale for anxiety; HAM-D: Hamilton rating scale for depression; NRS: Numeric Rating Scale; PSQI: Pittsburgh Sleep Quality Index; SF-MPQ: Short-form McGill Pain Questionnaire; WBMS: women with burning mouth syndrome.
